# Legal Restrictions on Practice by Quacks

**Published:** 1898-12-03

**Authors:** 


### LEGAL RESTRICTIONS ON PRACTICE BY QUACKS.

A legal correspondent of the *British Medical Journal* has dug up some ancient statutes which go far to show that none but qualified medical practitioners have the right to practise. So far as recent decisions go, it would seem that if an unregistered practitioner holds himself out to be what he really is—such, for instance, as M.D. of the Metropolitan College of New York—he cannot be properly convicted; whereas, if he assume the title of M.D. without further explanation, or any other title which would at once imply that his name was on the Register, he is liable to fine. It would seem, in fact, that it is the wilful and false assumption of such a title as shall mislead people to think he is on the Register that alone is punishable. As is well known, however, Mr. Horsley has maintained that it is registration under the Medical Act which alone gives the right to practise; that no right to practise exists except under the condition imposed by the Act, namely, that of registration; and that the practise of medicine or surgery by unregistered persons renders them liable to penalties. He holds that the Medical Act does far more than merely protect a title, and that, in fact, if it were properly put in force, the practise of medicine and surgery by quacks could be put a stop to. We wish we could think that Professor Horsley was right in regard to this. As we have remarked on several previous occasions, we fear that this conception of the scope of the Medical Acts is incorrect. According to the legal correspondent of the *British Medical Journal*, however, even in this case, even putting the most limited construction upon the scope of the Act, unregistered persons still have no right to practise, being debarred from so doing by ancient statutes. It would seem, indeed, that even before the days of Henry VIII. the Charter of the Royal College of Physicians of London gave them power to restrain all persons from practising the faculty of medicine in the City of London, or within seven miles thereof, without the College licence, under a penalty of £5; and by a statute in the reign of Henry VIII., which confirmed this charter, it was further enacted that "No person shall be suffered to exercise or practise in Physick through England" without a licence from the College, "except he be a graduate of Oxford or Cambridge which hath accomplished all things for his form without any grace," but it is to be noted that no mention is made of any penalty. Somewhat similar provisions were made as to the practise of surgery. Now it would appear that this statute has never been repealed, and thus that, while the Medical Act of 1858 imposed a penalty on all who falsely pretended to be, or assumed a title implying that they were registered, the right to practise either medicine or surgery was already restricted to those licensed by the Royal Colleges. Whether the judges would allow old statutes which have for so long lain dormant to be revived is a question which certainly should be decided. But this much is quite clear, that when we ask that some legal restriction should be placed upon the free and independent practise of medicine and surgery by quacks, we are not going against any principle of English law, for, indeed, the prohibition of such unqualified practise was the old principle on which these ancient statutes were founded, statutes which apparently remain unrepealed to this day.

